# Effects of a worksite labor gymnastics program on novice and experienced workers

**DOI:** 10.47626/1679-4435-2024-1242

**Published:** 2024-11-14

**Authors:** Jonhatan Norte Magno da Silva, Lucas Gomes Miranda Bispo, Carla Mirela Souza Silva, Wilza Karla dos Santos Leite, Elamara Marama Araujo Vieira, Tânia Daniela Felgueiras de Miranda Lima

**Affiliations:** 1 Universidade Federal de Alagoas, Campus do Sertão, Delmiro Gouveia, AL, Brazil; 2 Departamento de Engenharia de Produção e Transportes, Universidade Federal do Rio Grande do Sul, Porto Alegre, RS, Brazil; 3 Universidade Federal do Amapá, Macapá, AP, Brazil; 4 Universidade Federal da Paraíba, João Pessoa, PB, Brazil; 5 Departamento de Engenharia Eletromecânica, Universidade da Beira Interior, Covilhã, Portugal

**Keywords:** interpersonal relations, psychological well-being, labor gymnastics, occupational health, relações interpessoais, bem-estar psicológico, ginástica laboral, saúde ocupacional

## Abstract

**Introduction:**

Worksite labor gymnastics programs are interventions that can positively affect industrial workers. Studies show that experienced and novice workers perceive their working conditions differently. Our premise is that labor gymnastics programs affect these two groups diversely.

**Objectives:**

This study aimed to evaluate the benefits of an labor gymnastics program for experienced and novice industrial workers.

**Methods:**

Novice (476) and experienced (489) workers working for a footwear factory located in Northeastern Brazil participated in an labor gymnastics program for 5 months (October 2019 to February 2020). The variables musculoskeletal pain relief improved interpersonal relationships, willingness to work, psychophysiological well-being, and perceived difficulties in performing occupational tasks were assessed. Data were analyzed using an ordinal logistic regression model. The odds ratio modeled the relationship between the labor gymnastics program and the variables**. Results:** Experienced workers were six times more likely to report psychophysiological well-being, and novice workers were twice as likely to report improved interpersonal relationships. The benefits of the labor gymnastics program were different for experienced and novice workers.

**Conclusions:**

Although both groups can experience musculoskeletal pain relief, novice workers reported more commonly the variable improved interpersonal relationships, while experienced workers reported psychophysiological well-being. These findings help us better understand the outcomes of labor gymnastics programs according to length of service of industrial workers.

## Abstract (pt)

**Introdução:**

O programa de ginástica laboral é uma intervenção que pode afetar positivamente os trabalhadores industriais. Estudos indicam que existem diferenças na percepção das condições de trabalho entre trabalhadores experientes e novatos. Nossa premissa é que existe uma diferença no efeito de um programa de ginástica laboral entre esses dois grupos.

**Objetivos:**

Este estudo buscou avaliar os benefícios do programa de ginástica laboral no local de trabalho para trabalhadores industriais experientes e novatos.

**Métodos:**

Durante 5 meses (outubro de 2019 a fevereiro de 2020), trabalhadores novatos (476) e experientes (489) de uma fábrica de calçados localizada na região Nordeste do Brasil realizaram o programa de ginástica laboral. Foram avaliadas as variáveis alívio de dores musculoesqueléticas, melhora nas relações interpessoais, disposição para trabalhar, bem-estar psicofisiológico e dificuldades percebidas na execução das tarefas laborais. Os dados foram analisados por meio de modelo de regressão logística ordinal. A razão de chances modelou a relação entre o programa de ginástica laboral e as variáveis.

**Resultados:**

Os trabalhadores experientes tiveram uma chance seis vezes maior de relatar bem-estar psicofisiológico, e os trabalhadores novatos tiveram uma chance duas vezes maior de relatar melhora nas relações interpessoais. Os benefícios do programa de ginástica laboral se mostraram diferentes para trabalhadores experientes e novatos.

**Conclusões:**

Embora ambos os grupos possam experimentar alívio da dor musculoesquelética, a variável melhora nas relações interpessoais é mais comumente relatada pelos trabalhadores novatos, enquanto os trabalhadores experientes relatam melhora no bem-estar psicofisiológico. Essas descobertas auxiliam na melhor compreensão dos resultados de um programa de ginástica laboral de acordo com o tempo de experiência profissional dos trabalhadores industriais.

## INTRODUCTION

Ergonomics was developed based on the need for changes in the workplace to accommodate the characteristics of workers, even in changing scenarios.^1^ Ergonomic interventions include labor gymnastics programs (LGPs), which consist of physical exercises in the workplace.^2^ However, the effects of LGPs have been debated, as its effectiveness depends on the method implemented, the intended objective, and the psychophysiological characteristics of the organization and the workers.^3^

LGPs can be categorized as preparatory (the muscles and joints most required at work are prepared for strength, speed, or endurance), compensatory (an active pause at work to relax frequently used muscles and exercise poorly used muscles), and relaxing (seeking to remove muscle tension accumulated during the working day).^4^

The benefits of implementing LGPs include improved flexibility and motor coordination, the likelihood of a physically active lifestyle, increased confidence in interpersonal relationships, work capacity and perceived fatigue control, and changes in health behavior, postural habits, and reduced muscle tone in the trapezius region.^2,5-8^ Evidence also shows that LGPs prevents chronic pain and reduces the risk of developing musculoskeletal disorders and absenteeism.^9,10^

On the other hand, LGPs analysis should seek, whenever necessary, to group workers according to their sociodemographic, physiological, and occupational characteristics, and not only into case and control groups.^3^ When workers were sorted into different groups, results were observed for those who exercised at home and at the workplace,^6^ in men and women,^11^ and in white-collar and blue-collar workers.^12^ However, the literature looking at the influence of LGPs on workers with different degrees of occupational experience is scarce. Thus, workers can be grouped into two groups: novice and experienced, yet little is known about the benefits of LGPs for each group.^13^

Some studies have assessed novice and experienced workers. Denadai et al.^14^ found that novice workers had more reduced biomechanical exposure after educational training in ergonomics. As novices, they tend to have been exposed to lower biomechanical and stress demands, given that the length of service in the company is a known occupational risk factor.^15,16^ Experienced workers, in contrast, have greater variability in learned motor patterns, lower muscle electromyographic activity, and greater range of motion, which can help in building protective strategies at the workplace.^17^

Experienced workers perceive and observe hazards more easily, resulting in multiple accidents among novice workers.^18^ Experienced workers maintain a more neutral posture in physically demanding tasks.^19^ Therefore, novice workers should be trained in the proper use of noncontractile tissues, especially in the lower back.^20^ Although experienced workers are more productive and work more safely,^21^ their unfavorable habits can be difficult to change.

Therefore, the beneficial effects of worksite LGPs on novice and experienced workers may be contrasting. In light of the above, this article aims to assess the benefits of an LGP in two groups of industrial workers (novice *versus* experienced) within 5 months.

## METHODS

### OCCUPATIONAL SETTING

This study was conducted in a footwear factory that produces 272,500 pairs of footwear per day and employs 2,045 workers in Northeastern Brazil. The characteristics of work in the factory are similar to those found in many footwear factories in Brazil, requiring a great deal of manual activity and repetitive movements at high speed, keeping the upper limbs and trunk in an inadequate position and exerting force for a large part of the working day.^16^

### LGP DESIGN

The LGP was designed, implemented, and monitored by five physical educators over 5 months (October 2019 to February 2020). In previous similar studies, LGPs lasted 4 months on average.^10^ The LGP implemented was compensatory and lasted 10 minutes,^2,6,11,22^ including a warm-up (2 minutes), stretching (6 minutes), and relaxation (2 minutes) activity. Experienced and novice workers performed the same exercise protocol. The goal was to pause the work activity in the middle of the day, allowing workers to exercise underused muscles and tendons (antagonists) and relax frequently used muscles (agonists), helping to correct postures and gain flexibility.^4^ LGPs involve the upper and lower limbs and the back, as the demands of work in the footwear industry can require different parts of the body to work.^16^

During the LGP, balls of various sizes, medicine balls, elastic bands, hand massagers, bars, and audiovisual materials were used. The warm-up included walking with alternating and synchronized movements on the lower and upper limbs. Stretching exercises included a series of three repetitions for the back and lower and upper limbs. Relaxation involved breathing exercises and massage therapy.

### STUDY SAMPLE

The sample of novice workers was selected from the 476 newly hired workers (with only 1 or 2 days on the job).^14,17,21^ All novice workers agreed to participate in the study. On the other hand, experienced workers eligible for the study were those with more than 1 year of experience.^21^ All 554 experienced workers were invited to participate in the study, and 489 (88.27%) agreed.

The exclusion criteria were workers < 18 years of age; absence from work during the 5 months of the study; hypertension; diabetes; history of occupational accidents or disease; or pregnancy. A total of 458 (96.22%) novice workers and 456 (82.31%) experienced workers met the criteria. The sample consisted of 914 industrial workers.

Briefly, eight groups were formed: four for novices (G1, G2, G3, and G4) and four for experienced workers (G5, G6, G7, and G8). Participants were grouped based on the number of days per week they agreed to participate in the LGP: (i) groups 1 (G1) and 5 (G5), consisting of novices and experienced workers, respectively, did not perform the LGP, but paused their work activities for the 10 minutes; (ii) groups 2 (G2) and 6 (G6), consisting of novices and experienced workers, respectively, participated in the protocol 2 days per week; (iii) groups 3 (G3) and 7 (G7), consisting of novices and experienced workers, respectively, participated in the protocol 4 days per week; and (iv) groups 4 (G4) and 8 (G8), consisting of novices and experienced workers, respectively, participated in the protocol 5 days per week.

### SURVEY INSTRUMENT

As the sample was defined, a questionnaire by Silva et al.^3^ was administered. The dependent variables collected were musculoskeletal pain relief, improved interpersonal relationships, willingness to work, psychophysiological well-being, and perceived difficulties in performing occupational tasks. The answers were dichotomized as yes or no. Age, sex, and body mass index (BMI) were also assessed. Workers’ BMI was categorized according to the World Health Organization (WHO) classification.^23^ An adapted version of the Nordic Questionnaire of Musculoskeletal Symptoms (QNSO)^24^ was used to identify musculoskeletal symptoms in the past 7 days. The Brazil Research Ethics Committee approved the research project; Certificado de Apresentação de Apreciação Ética (Certificate of Submission for Ethical Appraisal) No. 61602616.6.0000.0121.

### STATISTICAL ANALYSIS

Cronbach’s alpha coefficient was used to analyze the reliability of the dependent variables. Multicollinearity between the dependent variables was analyzed using the variance inflation factor (VIF). Descriptive statistics were used to characterize the participants. The chi-square test was used to verify the homogeneity of the responses and the characteristics of the industrial workers who formed the groups. The Mann-Whitney test was used to compare symptoms between novice and experienced workers. The tests used a significance level of 5%.

The association between the dependent variables was assessed using Cramér’s V coefficient. The odds ratio (OR) estimator, extracted from ordinal logistic regression models, sought to verify the influence of the frequency of LGP (independent variable) on the dependent variables. Leverage points (influential and inconsistent observations) were extracted from the regression models.^25^ The accuracy of the regression models was used as a metric of accuracy. All statistical procedures were conducted with R.^26^

## RESULTS

The data were collected and the mathematical procedures were performed. Cronbach’s alpha coefficient was equal to 0.65 and 0.67 for the data from novice and experienced workers, respectively. The highest VIF was equal to 1.95 between the factors musculoskeletal pain relief and improved interpersonal relationships (among experienced workers). These results show a moderate internal consistency for the data,^27^ and no multicollinearity between the dependent variables.^28^ The length of service of experienced workers was on average 6.01 ± 4.8.

### SAMPLE CHARACTERISTICS

Table 1 shows the characteristics of the industrial workers and their responses to the questionnaire. A greater number of industrial workers (among those who decided to participate in the LGP) showed musculoskeletal pain relief, improved interpersonal relationships, improved psychophysiological well-being, and a greater willingness to work.

**Table 1:** Assessment of the characteristics and responses of experienced and novice workers

Variables	Experienced workers					Novice workers					Experienced workers versus novice workers			
	G5	G6	G7	G8		G1	G2	G3	G4		G1 x G5	G2 x G6	G3 x G7	G4 x G8
	n (%)	n (%)	n (%)	n (%)	p-value	n (%)	n (%)	n (%)	n (%)	p-value	p-value	p-value	p-value	p-value
Sex														
Female	13 (37.14)	34 (40.96)	32 (44.44)	126 (47.37)	0.566	6 (33.33)	13 (37.14)	29 (4.28)	146 (4.84)	0.701	1.000	0.856	0.736	0.436
Male	22 (62.86)	49 (59.04)	40 (55.56)	140 (52.63)		12 (66.67)	22 (62.86)	43 (59.72)	187 (56.16)					
BMI														
Underweight	0 (0.00)	2 (2.41)	2 (2.78)	6 (2.56)	0.859	1 (5.56)	0 (0.00)	3 (4.17)	30 (9.00)	**0.013**	0.165	0.692	0.606	**0.001**
Normal weight	20 (57.14)	50 (60.24)	48 (66.67)	155 (58.27)		8 (44.44)	19 (54.29)	45 (62.50)	211 (63.37)					
Overweight	12 (34.29)	23 (27.71)	17 (23.61)	88 (33.08)		4 (22.22)	13 (37.14)	16 (22.22)	75 (22.52)					
Obesity 1	3 (8.57)	8 (9.64)	4 (5.55)	15 (5.64)		4 (22.22)	3 (8.57)	6 (8.34)	15 (4.50)					
Obesity 2	0 (0.00)	0 (0.00)	0 (0.00)	2 (0.75)		1 (5.56)	0 (0.00)	2 (2.77)	2 (0.61)					
Age (years)														
15-19	0 (0.00)	0 (0.00)	0 (0.00)	3 (1.13)	0.103	1 (5.56)	2 (5.71)	4 (5.56)	24 (7.21)	**0.012**	**0.014**	0.060	0.180	**0.001**
20-29	12 (34.29)	39 (46.99)	43 (59.72)	155 (58.27)		11 (61.11)	21 (60.00)	44 (61.11)	207 (62.16)					
30-39	16 (45.71)	31 (37.35)	24 (33.33)	83 (31.20)		2 (11.11)	6 (17.14)	18 (25.00)	79 (23.72)					
40-49	7 (20.00)	10 (12.05)	4 (5.56)	22 (8.27)		2 (11.11)	4 (11.43)	6 (8.33)	18 (5.40)					
> 50	0 (0.00)	3 (3.61)	1 (1.39)	3 (1.13)		2 (11.11)	2 (5.72)	0 (0.00)	2 (0.61)					
MPR														
No	9 (25.71)	22 (26.51)	16 (22.22)	21 (7.89)	**0.000**	11 (61.11)	9 (25.71)	14 (19.44)	40 (12.01)	**0.000**	**0.026**	1.000	0.837	0.129
Yes	26 (74.29)	61 (73.49)	56 (77.78)	245 (92.11)		7 (38.89)	26 (74.29)	58 (80.55)	293 (87.99)					
IIR														
No	25 (71.43)	19 (22.89)	8 (11.11)	26 (9.77)	**0.000**	10 (55.56)	10 (28.57)	10 (13.89)	34 (10.21)	**0.000**	0.396	0.674	0.801	0.968
Yes	10 (28.57)	64 (77.11)	64 (88.89)	240 (90.23)		8 (44.44)	25 (71.43)	62 (86.11)	299 (89.79)					
WTW														
No	26 (74.29)	12 (14.46)	13 (18.06)	23 (8.65)	**0.000**	10 (55.56)	4 (11.43)	10 (13.89)	28 (8.41)	**0.000**	0.283	0.885	0.649	1.000
Yes	9 (25.71)	71 (85.54)	59 (81.94)	243 (91.35)		8 (44.44)	31 (88.57)	62 (86.11)	305 (91.59)					
PWB														
No	25 (71.43)	12 (14.46)	4 (5.56)	10 (3.76)	**0.000**	9 (50.00)	5 (14.29)	8 (11.11)	25 (7.51)	**0.000**	0.216	1.000	0.366	0.078
Yes	10 (28.57)	71 (85.54)	68 (94.44)	256 (96.24)		9 (50.00)	30 (85.71)	64 (88.89)	308 (92.49)					
PD														
No	30 (85.71)	62 (74.70)	59 (81.94)	220 (82.71)	0.362	15 (83.33)	31 (88.57)	61 (84.72)	304 (91.29)	0.297	1.000	0.150	0.823	**0.002**
Yes	5 (14.29)	21 (25.30)	13 (18.06)	46 (17.29)		3 (16.67)	4 (11.43)	11 (15.28)	29 (8.71)					

Values in bold indicate significant differences between the characteristics or responses of industrial workers in different groups.

MPR = musculoskeletal pain relief; PWB = psychophysiological well-being; PD = perceived difficulties in performing occupational tasks; WTW = willingness to work; BMI = body mass index; IIR = improved interpersonal relationships.

**Figure 1:**
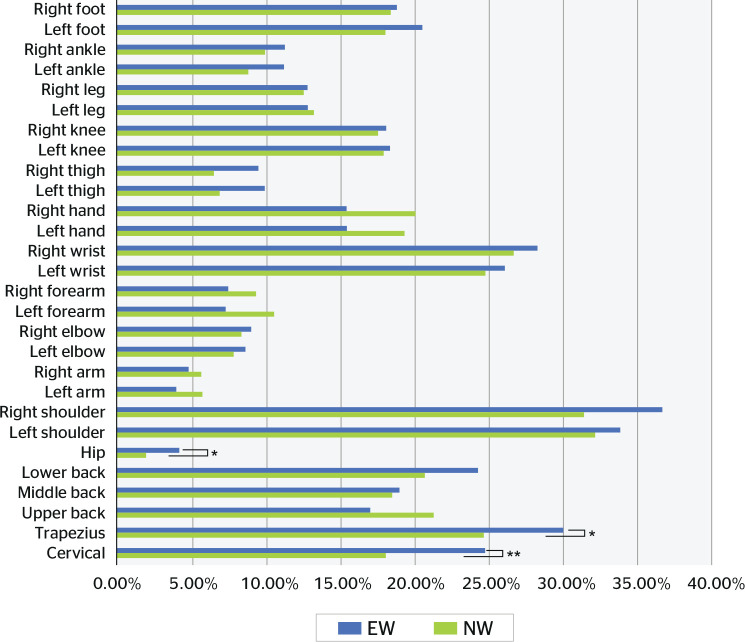
Musculoskeletal symptoms of experienced (EW) and novice workers (NW). Association between dependent variables. * < 0.05; ** < 0.01.

Figure 1 summarizes the symptoms experienced and novice workers reported. A greater number of experienced workers presented symptoms in the cervical, trapezius, and pelvic regions. Lacaze et al.^29^ observed that these symptoms tend to reduce with LGPs. However, only a few body parts had significantly reduced symptom intensity.

Table 2 shows the associations between the dependent variables. Except for the variable perceived difficulties in performing occupational tasks, all the other dependent variables were associated with at least one of the other variables. Therefore, an improvement or worsening in one dependent variable leads to a high probability of having an effect on another variable.

**Table 2:** Cramér’s V coefficient values for novice workers (NW) and experienced workers (EW)

Variables	1	2	3	4	5
	NW				
MPR	-	0.125	0.477^*^	0.397	0.353
PD	0.074	-	0.004	0.057	0.071
PWB	0.563†	0.098	-	0.597†	0.548†
WTW	0.522†	0.095	0.579†	-	0.534†
IIR	0.515†	0.096	0.633†	0.606†	-
	EW				

MPR = musculoskeletal pain relief; PWB = psychophysiological well-being; PD = perceived difficulties in performing occupational tasks; WTW = willingness to work; BMI = body mass index; IIR = improved interpersonal relationships.

* Moderate association; † strong association.

### FREQUENCY OF PHYSICAL EXERCISE AND PSYCHOPHYSIOLOGICAL AND SOCIAL FACTORS

Table 3 shows the results of the logistic regression model between the frequency of the LGP and the dependent variables. The LGP increased around twofold the chances of novice workers reporting musculoskeletal pain relief and improved interpersonal relationships. Among experienced workers, the LGP increased around three times the odds for those who reported musculoskeletal pain relief and six times for those who reported psychophysiological well-being. Considering the model with all industrial workers, the three factors (musculoskeletal pain relief, improved interpersonal relationships, and psychophysiological well-being) seem to influence both novice and experienced workers. When the groups were assessed separately, a realistic estimate could be obtained to more easily distinguish how LGP benefits these factors.

**Table 3:** Results of the ordinal logistic regression model

Variables	Novice workers (n = 458)				Experienced workers (n = 455^*^)				All workers (n = 907^**^)			
	OR	LL_OR_	UL_OR_	p-value	OR	LL_OR_	UL_OR_	p-value	OR	LL_OR_	UL_OR_	p-value
MPR	2.06	1.14	3.73	0.016	2.91	1.66	5.10	0.000	2.57	1.72	3.83	0.000
IIR	2.21	1.16	4.19	0.015	1.21	0.61	2.42	0.581	1.83	1.16	2.90	0.010
WTW	1.46	0.69	3.08	0.323	1.44	0.75	2.73	0.270	1.37	0.84	2.22	0.207
PWB	1.11	0.48	2.58	0.810	6.08	2.61	14.18	0.000	1.90	1.08	3.36	0.027
PD	0.71	0.38	1.34	0.292	1.13	0.70	1.83	0.620	0.81	0.56	1.18	0.282

** A total of seven leverage points were excluded from the model with all industrial workers, and

* one leverage point was excluded from the model with experienced workers. The accuracy of the regression models was 72.71%, 61.98%, and 65.16% for experienced workers, novice workers, and the sample of all workers, respectively.

MPR = musculoskeletal pain relief; PWB = psychophysiological well-being; PD = perceived difficulties in performing occupational tasks; WTW = willingness to work; LL_OR_ = lower limit of the odds ratio; UL_OR_ = upper limit of the odds ratio; IIR = improved interpersonal relationships.

## DISCUSSION

This study assessed the benefits of a worksite LGP on novice and experienced workers on the variables musculoskeletal pain relief, psychophysiological well-being, perceived difficulties in performing occupational tasks, willingness to work, and improved interpersonal relationships. As a result, it was clear that novice and experienced workers had a greater chance of musculoskeletal pain relief after 5 months of the LGP. Improved psychophysiological well-being and improved interpersonal relationships were also observed on novice workers and experienced workers, respectively (Table 3). This analysis allowed us to verify, with greater precision, the real effects of the LGP both on novice and on experienced workers. It is worth noting that this approach has hardly been studied with large samples.^11^

Novice and experienced workers who participated more frequently in the LGP were two and three times more likely, respectively, to have musculoskeletal pain relief. In addition, workers with a longer length of service may experience more intense musculoskeletal symptoms,^15^ thus providing more evident benefits for experienced workers. Previous studies have shown that greater worker participation in LGPs reduces musculoskeletal complaints.^3^ LGPs also improve muscle strength and flexibility in some parts of the body.^12^ Andersen et al.^22^ demonstrated the benefits of LGPs on workers who already suffered from chronic musculoskeletal pain, increasing vitality and improving the social climate in the workplace.

Novice workers were twice as likely to report improved interpersonal relationships. This is because LGPs provide a time for integration between workers, encouraging improved interpersonal relationships because some moves require assistance from other workers.^11^ Previous findings have also shown improved interpersonal relationships after taking part in LGPs.^3^ Therefore, LGPs at the workplace can facilitate cooperation and trust between coworkers, build social capital in work teams, and increase productivity.^6^

LGPs can influence factors associated with psychophysiological well-being, reducing symptoms associated with anxiety, stress, and depression.^6^ It also improves physical fitness, reduces sedentary behavior, and enhances cardiometabolic health indices.^30^ It is worth noting that LGPs help individuals to introduce healthy habits.^8^ In addition, workers can expect improved motor skills, better control of fatigue, and improved work capacity.^2,7^ Genin et al.^13^ showed that experienced participants in an LGP had reduced fat mass and improved physical fitness. All these findings corroborate the results obtained in this study, in which experienced workers with greater participation in the LGP were six times more likely to report psychophysiological well-being.

In conclusion, this study revealed that LGPs have different benefits for novice and experienced workers. It is worth noting that LGPs are a set of exercises that provide a break from routine work, but do not reduce exposure to risk factors.^11^

This study has some limitations, as expected: (i) the data collected are based only on the perceptions of industrial workers; (ii) the independent variables could have been converted from a linguistic response scale to a more precise scale through a process of fuzzification and defuzzification; (iii) the physical effects were poorly explored; (iv) the sample size and financial limitations prevented preand post-tests for variables; (v) and the total length of service was not considered to group workers based on their level of experience during data analysis. Future studies should address these limitations.

## CONCLUSIONS

Different benefits were identified between novice and experienced workers 5 months from implementing the LGP. The weekly participation in the LGP was a relevant factor, increasing the possibility of positive responses associated with the independent variables. Novice workers showed improved interpersonal relationships, and experienced workers improved psychophysiological well-being. Both novice and experienced workers reported greater musculoskeletal pain relief. Therefore, the benefits of an LGP for novice and experienced workers are not always the same. These findings help to better understand the results of LGPs according to the length of service of industrial workers.
